# Supplementary material for: Learning curve and surgical outcome of robotic assisted colorectal surgery with ERAS program
Source: Sci Rep. 2022 Nov 29;12:20566. doi: 10.1038/s41598-022-24665-w (PMC9709162; doi:10.1038/s41598-022-24665-w)
Supplement: Supplementary file 2 — Supplementary Information 2. [file 41598_2022_24665_MOESM2_ESM.zip › RAL Raw data-Ñ[▒K/082/Patho_ñw╝╨░O▒Kñσ.pdf]

臺中榮民總醫院 臺中市臺灣大道四段1650號 病理部活體組織切片 臨床醫學實驗及病理診斷中心 檢驗(查)報告

[redacted]

Pathologic diagnosis:
1. Colon, terminal ileum, and appendix, right hemicolectomy --- a. Intramucosal adenocarcinoma, moderately differentiated, arising in a tubulovillous adenoma, of ascending colon. The appendix, ileocecal valve, bilateral cut ends and mesocolic lymph nodes (0/17) are free of tumor. b. Tubular adenoma, multifoci, of the colon.
2. Omentum, omentectomy --- Unremarkable.

Prognostic and predictive factor:
1. Tumor size: 0.3 cm.
2. Depth of invasion: Intramucosal carcinoma, invasion of lamina propria.
3. Histologic grade: G2: Moderately differentiated.
4. Angiolymphatic invasion: Not identified.
5. Perineural invasion: Not identified.
6. Tumor deposits (discontinuous extramural extension): Not identified.
7. Macroscopic tumor perforation: Not identified.
8. Surgical Margins: a) Proximal Margin: Uninvolved by invasive carcinoma, distance of tumor from margin: 6.3 cm. b) Distal Margin:Uninvolved by invasive carcinoma, distance of tumor from margin: 7.7 cm. c) Radial or Mesenteric Margin: Uninvolved by invasive carcinoma, distance of tumor from margin: More than 1 cm.
9. Lymph node status: Uninvolved (0/17).
10. Extranodal involvement: Not identified.
11. Tumor regression grading S/P CCRT: No known presurgical therapy.
12. Type of polyp in which invasive carcinoma arose: Tubulovillous adenoma.
13. Additional pathologic findings: Adenoma(s).
14. Pathological TNM Stage: pTisN0 (According to the eighth edition, American Joint Committee on Cancer Staging Guidelines for Tumors).
15. TNM descriptors: Not applicable.

Gross description:
The specimen consists of 1) a segment of colon, 13.5 cm in length, and 4.8 cm in distal cut end circumference, and a segment of terminal ileum, 3.5 cm in length, and 4 cm in cut end circumference. An unremarkable appendix, 6.2 cm, is noted. Two pedunculated polypoid lesion, 1.7x0.8 cm and 1.3x0.8 cm, is noted 5.8 cm and 6.1 cm to the ileocecal valve, respectively. On cross section, no invasion is present in both polyps. Several tiny polyps, up to 0.2 cm in diameter, are seen in the distal portion of colon. The appendix, ileocecal valve, and both cut ends are free of lesion. 2) "omentum", a piece of yellow tan and soft adipose tissue, 18x5.4x1.8 cm. Representative part for section: A) colon cut end B,M) ileum cut end C) appendix D) ileocecal valve E-F) 1.7 cm polyp G) 1.3 cm polyp H) other tiny polyps J-L) specimen 2 N-Q) lymph nodes.

Note:
This case has been peer reviewed by two doctors.
#T-59420_2 #M-82633_2 2 1105 U0000F
#T-C4560_2 #M-09183_2 0 1105 000000
#T-59200_2 #M-09183_2 0 1105 000000

已電子簽章

警告：本單僅供醫師參考使用、不具任何申請效力！！
請病患妥為保管、若不慎遺失需自負個人資料外洩責任！
